# Has the time come to use near-infrared spectroscopy as a routine clinical tool in preterm infants undergoing intensive care?

**DOI:** 10.1098/rsta.2011.0261

**Published:** 2011-11-28

**Authors:** Gorm Greisen, Terence Leung, Martin Wolf

**Affiliations:** 1 Department of Neonatology, Rigshospitalet and University of Copenhagen, Blegdamsvej 9, Copenhagen 2100, Denmark; 2 Biomedical Optics Research Laboratory, Department of Medical Physics and Bioengineering, University College London, London, UK; 3 Biomedical Optics Research Laboratory, Division of Neonatology, University of Zurich, Zurich, Switzerland

**Keywords:** near-infrared spectroscopy, preterm, brain, cerebral oxygenation, neurodevelopmental impairment, randomized controlled trial

## Abstract

Several instruments implementing spatially resolved near-infrared spectroscopy (NIRS) to monitor tissue oxygenation are now approved for clinical use. The neonatal brain is readily assessible by NIRS and neurodevelopmental impairment is common in children who were in need of intensive care during the neonatal period. It is likely that an important part of the burden of this handicap is due to brain injury induced by hypoxia–ischaemia during intensive care. In particular, this is true for infants born extremely preterm. Thus, monitoring of cerebral oxygenation has considerable potential benefit in this group. The benefit, however, should be weighed against the disturbance to the infant, against the limitations imposed on clinical care and against costs. The ultimate way of demonstrating the ‘added value’ is by a randomized controlled trial. Cerebral oximetry must reduce the risk of a clinically relevant endpoint, such as death or neurodevelopmental handicap. We estimate that such a trial should recruit about 4000 infants to have the power to detect a reduction in brain injury by one-fifth. This illustrates the formidable task of providing first-grade evidence for the clinical value of diagnostic methods. Is it a window of opportunity for the establishment of a rational basis before another technology is added to an already overly complex newborn intensive care?

## 1. Clinical research using near-infrared spectroscopy in newborn infants

The first clinical research use of near-infrared spectroscopy (NIRS) was seen in newborn infants and was published in 1985. NIRS was rapidly called ‘promising’ because the newborn brain is readily accessible by NIRS owing to the thin scalp and skull of infants. Over the following 25 years, NIRS has contributed substantially [1] to the pathophysiological background of clinical neonatology. The question of whether the time has come to put NIRS into clinical neonatal practice remains.

## 2. Cerebral oximetry

NIRS allows direct measurement of brain oxygenation, cerebral oximetry, every second (real-time monitoring). This can be performed in a number of ways as reviewed elsewhere [2]. Cerebral oxygenation is expressed as a percentage (from 0% to 100%) and represents the haemoglobin–oxygen saturation in all sections of the vasculature and therefore is ‘venous weighted’. Cerebral oxygenation serves as a surrogate measure of cerebral blood flow and a direct indicator of brain ischaemia/hyperaemia as it represents the balance between blood flow and oxygen requirement. Cerebral oximetry has already been used extensively for research in newborn infants and in neonatal cardiac care [3].

## 3. Cerebral oximetry in clinical practice

One retrospective [4] and two randomized controlled trials [5,6] have examined the clinical value of cerebral oximetry during cardiac surgery and cardiopulmonary bypass. Although the result is not entirely clear, there is good evidence of a relation between cerebral hypoxia and post-operative complications and some indication of improved outcome in patients managed with access to cerebral oximetry readings. Thus, it is not surprising that such monitoring has been recommended [7]. Several instruments that provide oximetry are approved for clinical use and they are entering operating rooms and cardiac intensive care units.

## 4. The case for clinical use in neonatology

Newborn infants suffer from a wide range of cardiopulmonary complications related to pulmonary immaturity, birth injury and malformations that may compromise oxygenation and/or circulation.

In view of this, it is a problem that the blood pressure is of limited value as a clinical guide to treatment [8]. Although echocardiography can provide a wealth of data relevant to the intensive care of extremely preterm infants [9] and can be repeated as often as necessary, it is a formidable task to provide reliable echocardiography services on demand in all neonatal intensive care units. Additionally, the positive predictive value of the echocardiographically determined state of low circulatory flow in predicting brain injury is low [10] and interventions aimed at preventing states of low flow have not been shown to reduce brain injury. Currently, there is no method for monitoring organ blood flow suitable for clinical needs [11]. Therefore, cerebral oximetry is a candidate for a breakthrough diagnostic tool needed for the clinical management of circulatory insufficiency in the newborn.

## 5. The special case: the extremely preterm infant shortly after birth

The cardiovascular transition at birth is a particularly serious challenge for infants born more than 12 weeks preterm—called extremely preterm. In a preterm infant, immature lung function impedes the recruitment of the vascular bed of the lungs and the closure of the arterial duct is delayed. The immature myocardium has limited systolic and diastolic function and, as a result, regulation of organ blood flow is weak and vulnerable [12]. Currently, extremely preterm infants have an average mortality of 20 per cent [13] and approximately 10 per cent of survivors develop cerebral palsy and 25 per cent of survivors grow up with either cerebral palsy or low intelligence quotient. Such neurodevelopmental impairment is a major cause of reduced quality of life as well as increased medical care, rehabilitation and special education costs in this population.

There is a high risk of neurodevelopmental impairment in extremely preterm infants that can be caused by several types of brain injury and subsequent disturbance of brain development [14]. One important mechanism is hypoxia–ischaemia [15]; another is hyperoxia [16]. Hypoxia–ischaemia of the central white matter of the immature brain is often complicated by severe brain haemorrhage [17]. These haemorrhages are typically preceded by episodes of low blood pressure [18], low cardiac output [19] and low cerebral blood flow during the first 24 h following birth [20]. Thus, circulatory insufficiency during the first few days of life is a root cause of brain injury in extremely preterm infants and cerebral oximetry during these first critical days could potentially make a very significant difference.

## 6. The arguments against starting to use cerebral oximetry in extremely preterm infants

**Figure 1.**
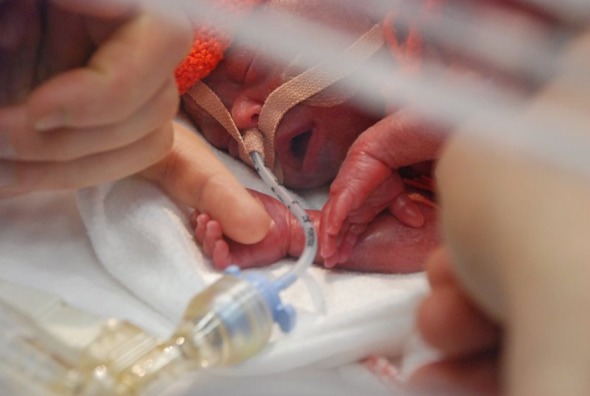
An infant shortly after birth born at 25 weeks of gestation. The tube for artificial ventilation passes through the nose to the trachea and is fixed to the skin with tape. The skin is fragile; hence this is acceptable only because ventilation is lifesaving. The cap covering the head and the sheet covering the body are partly to conserve heat, partly for the comfort of the baby, and partly to emphasize the dignity and individuality of the infant. In this context, the practical problem of applying a new probe for monitoring is a significant concern. (Online version in colour.)

The extremely preterm infant is small with fragile skin. Placing a probe on the head and maintaining its position is another challenge for nurses, and it interferes with the already compromised bonding between parents and infant (figure 1). It must have a clear benefit.

Cerebral oximetry during surgery is partly based on the use of trends, i.e. comparing the current value with the value before anaesthesia when the patient was awake, or at least before some critical manoeuvre, such as clamping of an artery. This in general does not make sense in the extremely preterm infant after birth. It cannot be assumed that any particular moment after stabilization of respiration and postnatal circulation can be used as a reference point.

It is therefore a specific limitation of the current commercial NIRS oximeters that the absolute level of oxygenation is relatively poorly determined. Reliability of the measurement compared with oxygen saturation in jugular venous blood is 8–9%. This corresponds to the limits of agreement ranging from −17 to +17 per cent [21]. While this may be sufficient for the detection of gross abnormality [22], such abnormalities are likely to be relatively rare and may often be detected by other means. More generally, these infants are already monitored and are under close clinical supervision, and, while existing means of judging the circulatory sufficiency are not ideal, they are not without value. Thus, the key question is whether cerebral oximetry *adds* *significantly* to the total clinical picture, i.e. whether cerebral oximetry has added diagnostic value.

It is necessary to have reference values in order to diagnose cerebral hypoxia (or hyperoxia). Animal experimentation shows that cerebral oxygenation has to decrease to 35 per cent before electrical failure occurs, and that neuronal injury occurs if the low value persists for several hours [23,24].

For a diagnosis to be useful to the patient and improve clinical outcome, it is necessary that effective treatment is available. In some contexts, this is very likely to be the case for extremely preterm infants. Inadvertent mechanical hyperventilation induces cerebral vasoconstriction and decreased cerebral blood flow [25]. Inadvertent hyperventilation is strongly associated with cerebral white matter damage and cerebral palsy in surviving preterm infants [15]. Monitoring of the arterial carbon dioxide concentration by the transcutaneous route is possible, but not easy, and is therefore not universally used. Cerebral oximetry is likely to be an effective means of avoiding this problem simply by prompting the clinician to reduce ventilatory support. On the other hand, a state of low cardiac output is also common in extremely preterm infants [19]. Low cardiac output has significant long-term consequences [26] but it is difficult to treat [27].

A state of cerebral hypoxia will lead the clinician to consider allowing the arterial *p*CO_2_ to increase, to use vasopressors to increase the arterial blood pressure, to use volume transfusion or inotropes to increase the cardiac output, to give a red blood cell transfusion to increase blood haemoglobin and to increase the inspired oxygen concentration to increase arterial oxygen saturation.

A state of cerebral hyperoxia, on the other hand, may lead the clinician to attempt to reduce the arterial *p*CO_2_ or reduce the inspired oxygen fraction. Hyperoxygenation, oxygen toxicity and free oxygen radical damage are also implicated in brain white matter injury in extremely preterm infants [14].

Finally, cost is a significant concern. Healthcare services are stressed by increasing demands, increasing technical possibilities and increasing costs. Public or private, the resources used for healthcare cannot be used for other significant purposes, such as education, social security, protection of the environment and local, national or global law and justice. These are all important factors for the future of children. This emphasizes the need to be concerned with cost-effectiveness.

## 7. The case for formal evaluation of the clinical benefit of cerebral oximetry in extremely preterm infants

**Figure 2.**
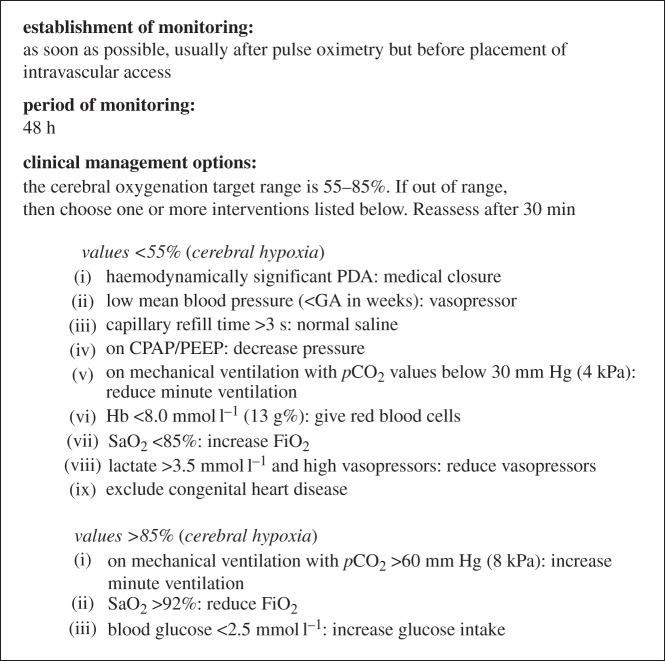
A clinical management guideline using cerebral oxygenation from the brain to improve the clinical care of extremely preterm infants during the first 48 h after birth. Many aspects of neonatal intensive care are involved. Although the guideline reflects a large body of knowledge of pathophysiology in the newborn brain, some of the interventions have not been shown directly to improve cerebral oxygenation. The guideline was developed during the planning of a randomized controlled trial by a European Academic Consortium with partners from Copenhagen, Utrecht, Leuven, Madrid, Zurich, Uppsala, Milan, Graz, Cambridge, Witten/Herdecke, Lyon and Cork. CPAP, continuous positive airway pressure; GA, gestational age; HB, haemoglobin; PDA, patent ductus arteriosus; PEEP, positive end-expiratory pressure.

Although all of the interventions described above have considerable pathophysio- logical rationality, they also have side-effects and may have unforeseen effects in a complex clinical context. On the other hand, a ‘reassuring’ reading of the cerebral oximeter may allow the clinician to defer an intervention that also has costs and may also have unwanted side-effects. Therefore, the only way to make sure that cerebral oximetry does more good than harm is to test it in combination with a physiology-based treatment guideline (figure 2).

Several measures of success can be defined. First, is it possible to reduce the amount of time spent with cerebral oxygenation out of target range? Second, is it possible to reduce molecular or electroencephalographic markers of acute hypoxic–ischaemic brain injury? Third, is it possible to reduce brain damage as determined by ultrasound or magnetic resonance imaging when the infant reaches term age and is ready to be discharged home? Finally, is it possible to reduce the risk of neurodevelopmental deficit as the child grows up? Although the first three measures are relevant, and even can be mechanistically necessary intermediate steps, it is only the last measure that is truly clinically meaningful.

## 8. A randomized controlled trial

The gold standard method for evaluating the clinical benefits of medical interventions is the randomized controlled trial. Apart from randomization, this methodology is based on strict inclusion and exclusion criteria on a single prespecified primary ‘endpoint’, and on a sample size that is able to exclude a minimal clinically relevant difference in this endpoint between the experimental and the control groups. This means that the trial should be able to give statistical support for a clinically relevant effect size.

## 9. Outline of a trial in extremely preterm infants testing the effect of cerebral oximetry on neurodevelopment at 2 years of age

**Figure 3.**
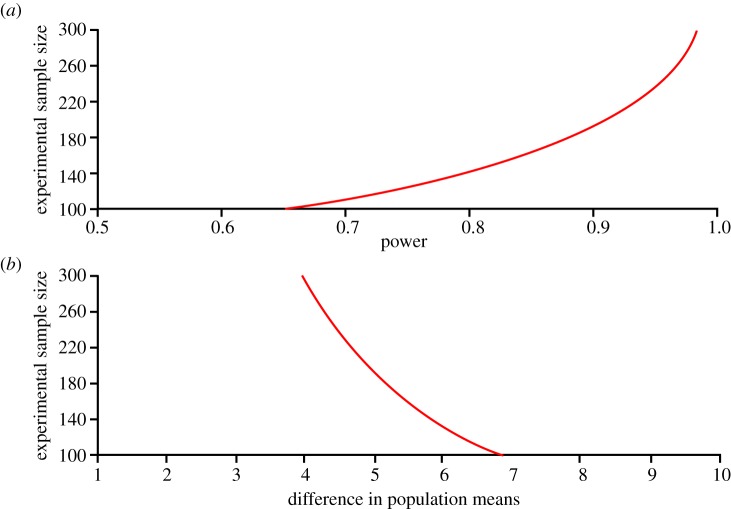
Calculation of sample size for a randomized controlled trial to demonstrate the clinical benefit of cerebral oximetry in extremely preterm infants. It is hoped that the risk of brain injury can be reduced and it is expected that this will result in improved cognitive development. Calculations are based on an effect size of five points, corresponding to a Cohen *d* of 0.33, as the standard deviation of a test measuring cognitive function is 15 points, and a study power of 90%. It can be seen that 190 infants will be needed in each of the groups and that the groups must be even larger if the effect size is less, or if the study power should be higher. (Online version in colour.)

Entry criteria will be hospital-born, more than 12 weeks preterm, a clinical decision of full life support and parental informed consent. The monitoring by cerebral oximeter must be started before 3 h of age. To detect a difference of five points in the primary outcome (s.d. 15 points) with a chance of 90 per cent at the 5 per cent significance level, it is necessary to have outcome data on 190 infants in each group (figure 3). As mortality in these infants is as high as 20 per cent, randomization of 250 infants to each intervention group is needed.

This plan, however, assumes that cerebral oximetry may have no effect on mortality. Although it is not predicted that monitoring the brain and optimizing brain oxygenation will change mortality, this is not certain. Therefore, mortality should be included in the primary endpoint, and most randomized controlled trials on early interventions in extremely preterm infants actually do have a combined endpoint, such as death or neurodevelopmental impairment. As the risk of neurodevelopmental impairment is 25 per cent and a reasonable benefit of cerebral oximetry to be hoped for would be a reduction to 20 per cent, the number needed to be randomized will be 4100 infants.

## 10. Is an adequately powered randomized controlled trial feasible?

Many neonatologists have great expectations for cerebral oximetry at the present time and are prepared to start moving. At the same time, there is sound scepticism, as neonatology is a clinical field with a history of significant iatrogenic epidemics. The best known examples are oxygen-induced retinopathy and postnatal steroid-induced cerebral palsy. Therefore, a well-constructed and well-conducted randomized clinical trial could help to promote the field of cerebral monitoring in a rational way. It is feasible, as 25 000 extremely preterm infants are born in Europe alone every year. It is evident, however, that an open-label trial of a several-day-long intervention is difficult to carry out well. Furthermore, a reduction in cerebral oxygenation outside the target range can only be achieved if the treatment guideline is efficient. A reduction in brain injury will be achieved only if the target range is physiologically meaningful, and if hypo- or hyperoxygenation in the first days of life constitutes a significant aetiological fraction. Finally, a reduction in neurodevelopmental deficit will be achieved only if other kinds of brain injury are not increased by the intervention.

## 11. Can instrumentation be improved for the extremely preterm infant?

**Figure 4.**
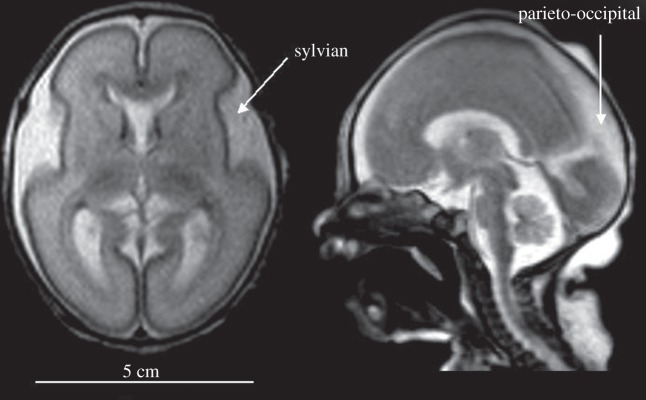
Magnetic resonance image of the brain shortly after birth in an infant born at 25 weeks of gestation. The brain is immature with few sulci and gyri. The thickness of both the skin and skull is 3–4 mm each. The sylvian fissure is widely open, and there is a large cerebrospinal fluid space in the parieto-occipital region. The fluid spaces constitute significant optical inhomogeneities for near-infrared spectroscopy, because photons can travel far with little chance of absorption or scattering. The result can be large differences in the values of cerebral oxygenation depending on the exact positioning of the source and the detector (MR images courtesy of M. Rutherford, Imperial College, London, UK).

**Figure 5.**
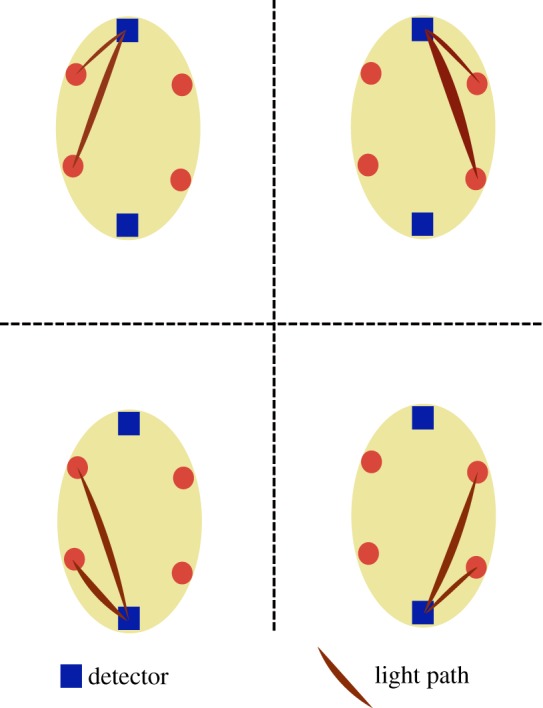
The layout of a probe with four sources and two detectors allowing simultaneous measurement of StO_2_ from four adjacent brain areas. This design may be used for imaging purposes; for the purpose of intensive care, the mean value of StO_2_ may be used as a global mean. It is less susceptible to imprecision owing to tissue heterogeneity compared with recording from a single brain area. Furthermore, the agreement among the four measures may be used to indicate the reliability of the mean value at any given time. (Online version in colour.)

The precision of current instruments is only 4–6% [28,29]. In the case of extremely preterm infants, the brain is immature and the cerebrospinal fluid space is wide in some regions (figure 4). This may lead to severe deviations from the assumption of optic homogeneity in terms of scatter and absorption and this in turn may significantly disturb the calculation of tissue oxygenation (StO_2_). One way to address this problem is to use multiple pathways, and such a probe is shown in figure 5. This design reduced the imprecision of measurement (measured as reproducibility of multiple re-settings of the probe on the head of preterm newborns) to 2.7 per cent [30].

### (a) Minimal source–detector separation: Monte Carlo simulations

Simulations were performed to search for a minimally reliable source–detector distance using a simple two-layer slab geometry, i.e. the skin/scalp layer and the brain. The optical and physiological parameters were those typical of a neonate with a gestational age of 30–31 weeks. It is known that the StO_2_ measurement based on spatially resolved spectroscopy (SRS) [31] is influenced by both the skin/scalp layer and the brain layer but to a different extent. Seven designs were analysed. They are based the distances of separation between one detector and two sources (table 1).

**Table 1.** Source–detector separation used for the Monte Carlo simulation, each set represents a potential design for an NIRS sensor optimized for the preterm newborn brain (see figure 5).

design	distance *c* (cm)	distance *d* (cm)
1	0.7	1.1
2	0.8	1.3
3	1.1	1.8
4	1.3	2.0
5	1.5	2.5
6	2.0	3.0
7	2.5	3.5

**Figure 6.**
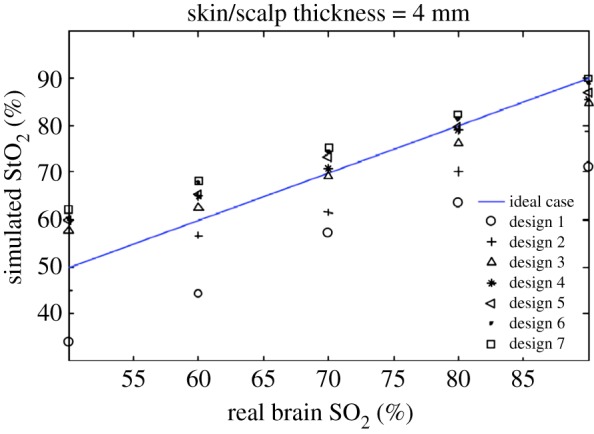
Simulated StO_2_ in a two-layer geometry as a function of brain SO_2_. The SO_2_ in the skin/skull layer was fixed at 70%. All results deviate from the true brain value. The discrepancy is expected for two main reasons. The first is that the spatially resolved spectroscopy (SRS)-based StO_2_ algorithm ignores the effect of water. The second reason is that the SRS algorithm is based on the assumption of a homogeneous semi-infinite half-space geometry. All designs are responsive to changes in brain SO_2_. Designs 1 and 2 underread greatly, whereas the designs with larger source–detector distance all produced similar results. (Online version in colour.)

**Figure 7.**
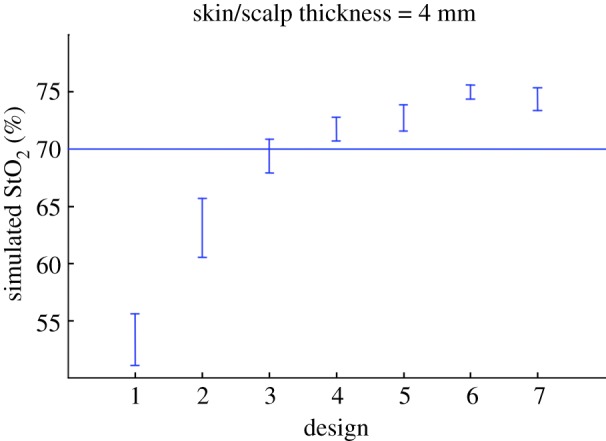
The sensitivity of the seven designs against SO_2_ changes in the skin/scalp layer as indicated by the vertical bars. Brain SO_2_ was fixed at 70% while the skin/scalp SO_2_ was varied between 50% and 90%. Simulated StO_2_ values from the two-layer geometry are influenced by the changes in the skin/scalp layer. In particular, designs 1 and 2 are not robust. (Online version in colour.)

From figures 6 and 7, we concluded that it appears to be possible to use shorter distances in the extremely preterm infant brain than those used by current general purpose instruments, and yet obtain a good compromise for the probe size, the sensitivity to the brain and the robustness against SO_2_ changes in the skin/scalp.

## 12. What are the alternatives?

Very few examples of diagnostic methods exist where the clinical benefit has been demonstrated in randomized trials. Most diagnostic methods are part of a more or less complex clinical context where gradual progress occurs over time with improving clinical outcomes. The technologies are exposed to the economical pressure of competition of providers and the competition of alternative use of healthcare resources.

On the one hand, cerebral oximetry can potentially become inexpensive as it is based on technology that can be mass produced. Also, the probe may be miniaturized and integrated with the electronics into a soft ‘plaster’ that may stick to the skin of the head of tiny infants and need little attention. Solid evidence of benefit to patients will create a large market. Evidence of benefit of an instrument using public domain technology can serve as a platform for healthy competition on user-friendliness and price.

On the other hand, what will happen if the clinical use of cerebral oximetry is not developed in a rational, evidence-based format? Then it may become another randomly applied expensive technology. Cerebral oximetry will be supported by anecdotal evidence, expert opinion, active branding and marketing. The consequences include unnecessary disturbances and risks to a very vulnerable group of patients and depletion of scarce healthcare resources.
